# Poly[hemi(ethyl­enediammonium) [di-μ-oxalato-indium(III)] dihydrate]

**DOI:** 10.1107/S1600536809008381

**Published:** 2009-03-14

**Authors:** Qiaozhen Sun, Yang Liu, Hongwu Li, Zhi Luo

**Affiliations:** a Department of Materials Chemistry, School of Materials Science and Engineering, Key Laboratory of Non-ferrous Metals of the Ministry of Education, Central South University, Changsha 410083, People’s Republic of China

## Abstract

In title compound, {(C_2_H_10_N_2_)_0.5_[In(C_2_O_4_)_2_]·2H_2_O}_*n*_, the unique In^III^ ion is coordinated by eight O atoms from four oxalate ligands in a distorted square-anti­prismatic environment. The doubly bis-chelating oxalate ligands act as bridging ligands connecting symmetry-related In^III^ ions and forming a three-dimensional open framework structure. Ethyl­enediammonium cations and water mol­ecules occupy the voids within the structure. The unique ethyl­enediammonium cation and one water mol­ecule both lie on a twofold rotation axis. One of the other two water mol­ecules residing on general crystallographic sites was refined as disordered with half occupancy. In the crystal structure, cations and water mol­ecules are linked to the anionic framework *via* inter­molecular O—H⋯O and N—H⋯O hydrogen bonds.

## Related literature

For background information on open-framework materials, see: Fang *et al.* (2004 ▶); Li *et al.* (2008 ▶); Serre *et al.* (2006 ▶); Sun *et al.* (2006 ▶). For related materials containing the oxalate ligand, see: Audebrand *et al.* (2001 ▶, 2004 ▶); Kokunov *et al.* (2004 ▶); Stock *et al.* (2000 ▶); Chakrabarti & Natarajan (2002 ▶); Evans & Lin (2001 ▶); Vaidhyanathan *et al.* (2001 ▶); Gavilan *et al.* (2007 ▶); Bataille *et al.* (2000 ▶); Trombe *et al.* (2001 ▶); Yuan *et al.* (2004 ▶). For indium oxaltes, see: Audebrand *et al.* (2003 ▶); Bulc *et al.* (1983 ▶); Bulc & Golič (1983 ▶); Chen *et al.* (2003 ▶); Huang & Lii (1998 ▶); Jeanneau *et al.* (2003 ▶); Yang *et al.* (2005 ▶); For the bond-valence method, see: Brown (1996 ▶). For bond distances and angles for bridging bidentate oxalate groups, see: Hann (1957 ▶).
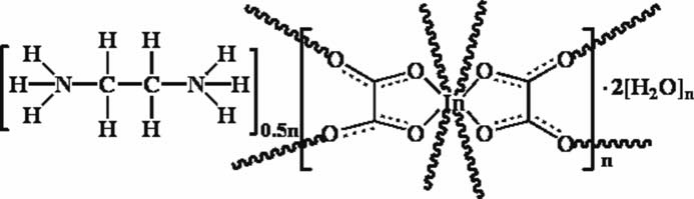


## Experimental

### 

#### Crystal data

(C_2_H_10_N_2_)_0.5_[In(C_2_O_4_)_2_]·2H_2_O
*M* *_r_* = 357.95
Orthorhombic, 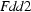

*a* = 15.8498 (4) Å
*b* = 31.1643 (8) Å
*c* = 8.6618 (2) Å
*V* = 4278.48 (18) Å^3^
*Z* = 16
Mo *K*α radiation
μ = 2.26 mm^−1^
*T* = 293 K
0.40 × 0.38 × 0.38 mm

#### Data collection

Bruker SMART CCD diffractometer
Absorption correction: multi-scan (*SADABS*; Bruker, 2001 ▶) *T*_min_ = 0.426, *T*_max_ = 0.467 (expected range = 0.387–0.424)
7189 measured reflections
1679 independent reflections
1673 reflections with *I* > 2σ(*I*)
*R*_int_ = 0.025

#### Refinement

*R*[*F* ^2^ > 2σ(*F* ^2^)] = 0.018
*wR*(*F* ^2^) = 0.046
*S* = 1.06
1679 reflections
160 parameters
13 restraints
H-atom parameters constrained
Δρ_max_ = 0.45 e Å^−3^
Δρ_min_ = −0.71 e Å^−3^
Absolute structure: Flack (1983 ▶), 668 Friedel pairs
Flack parameter: 0.00 (3)

### 

Data collection: *SMART* (Bruker, 2001 ▶); cell refinement: *SAINT* (Bruker, 2001 ▶); data reduction: *SAINT*; program(s) used to solve structure: *SHELXTL* (Sheldrick, 2008 ▶); program(s) used to refine structure: *SHELXTL*; molecular graphics: *SHELXTL*; software used to prepare material for publication: *SHELXTL*.

## Supplementary Material

Crystal structure: contains datablocks global, I. DOI: 10.1107/S1600536809008381/lh2779sup1.cif

Structure factors: contains datablocks I. DOI: 10.1107/S1600536809008381/lh2779Isup2.hkl

Additional supplementary materials:  crystallographic information; 3D view; checkCIF report

## Figures and Tables

**Table 1:** Hydrogen-bond geometry (Å, °)

*D*—H⋯*A*	*D*—H	H⋯*A*	*D*⋯*A*	*D*—H⋯*A*
N1—H1*C*⋯O*W*1^i^	0.89	2.35	2.880 (8)	118
N1—H1*B*⋯O7^ii^	0.89	2.47	2.956 (5)	115
N1—H1*B*⋯O*W*3	0.89	2.21	2.825 (8)	126
N1—H1*C*⋯O4^i^	0.89	2.44	3.140 (6)	136
N1—H1*C*⋯O5^iii^	0.89	2.38	3.166 (5)	147
O*W*1—H*W*1*A*⋯O2^iv^	0.85	2.04	2.889 (5)	180
O*W*2—H*W*2*B*⋯O1^iii^	0.85	2.46	3.241 (15)	153
O*W*2—H*W*2*A*⋯O3	0.85	2.39	3.12 (3)	145
O*W*3—H*W*3*B*⋯O8^v^	0.85	2.19	2.870 (6)	137
O*W*3—H*W*3*A*⋯O7	0.85	2.26	2.971 (7)	141
O*W*3—H*W*3*A*⋯O3^ii^	0.85	2.40	2.962 (6)	124

Symmetry codes: (i) 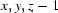; (ii) 
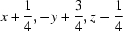
; (iii) 
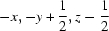
; (iv) 
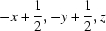
; (v) 
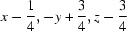
.

**Fig. 1.**
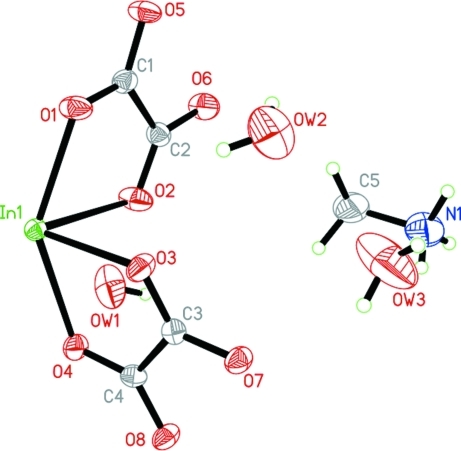
The asymmetric unit showing the atom-numbering scheme. Displacement ellipsoids are drawn at the 50% probability level.

**Fig. 2.**
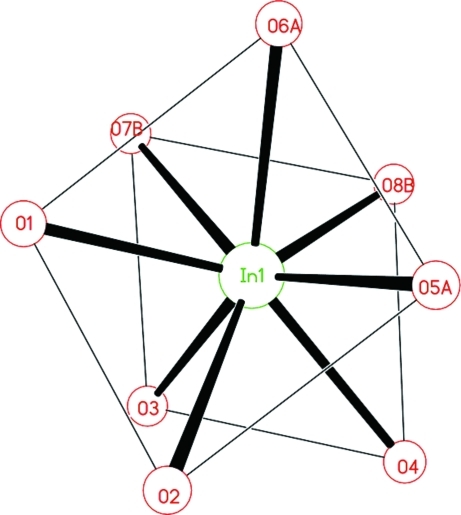
The distorted square antiprismatic environment of Indium. Symmetry codes A: -x, 0.5 - y, 1/2 + z; B: x - 1/4, 0.75 - y, 1/4 + z.

**Fig. 3.**
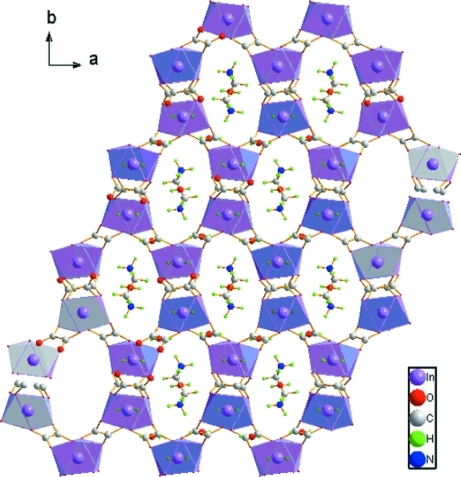
Part of the crystal structure viewed crystal along the c axis, the ethylenediammonium and water molecules reside in the voids.

## supplementary crystallographic information

### Comment

The synthesis of open-framework materials has emerged as an important area of research because of their potential applications in separation processes, ion exchange and catalysis. In the past few years, there has been considerable effort in designing open-framework structures formed by metal organic carboxylates because of its interesting structural features and the quality for apt design (Fang *et al.*, 2004; Li *et al.*, 2008; Serre *et al.*, 2006; Sun *et al.*, 2006) of which the oxalate ligand plays a major role in the assembly of metal-organic porous frameworks. Many metal oxalate structures are reported such as tin (Audebrand *et al.*, 2001; Kokunov *et al.*, 2004; Stock *et al.*, 2000), zinc (Chakrabarti & Natarajan, 2002; Rvans & Lin, 2001; Vaidhyanathan *et al.*, 2001), zirconium (Audebrand *et al.*, 2004; Gavilan *et al.*, 2007), rare earth (Bataille *et al.*, 2000; Trombe *et al.*, 2001; Yuan *et al.*, 2004). The structures of these compounds vary from monomers, dimmers, chains, layered honeycomb networks to three dimensional frameworks. In this paper, we selected indium and synthesized the three-dimensional indium oxalate compound [(C_2_N_2_H_10_)_0.5_In(C_2_O_4_)_2_.2H_2_O]_n_ (Fig. 1). Although many indium oxalates have been reported (Audebrand *et al.*, 2003; Bulc *et al.*, 1983; Chen *et al.*, 2003; Huang & Lii, 1998; Jeanneau *et al.*, 2003; Yang *et al.*, 2005), relatively a few of them are three dimensional open frameworks (Chen *et al.*, 2003; Huang & Lii, 1998; Yang *et al.*, 2005).

In the title structure, the In ion is coordinated by eight O atoms from four tetradentate oxalate groups, forming a distorted square antiprismatic arrangement (Fig. 2) in which atoms O1, O2, O5A and O6A (Symmetry code A: -*x*, 0.5 - *y*, 1/2 + *z*) are approximately in the same plane with a deviation of ca. 0.01 Å, while the other plane (formed by atoms O3, O4, O7B and O8B; Symmetry code B: *x* - 1/4, 0.75 - *y*, 1/4 + *z*) is significantly distorted, with a deviation of ca. 0.24 Å. The eight In—O bond distances vary between 2.168 (3) and 2.423 (3) Å (average 2.279 Å), which agrees well with the value 2.265 Å calculated for an eightfold coordinated indium atom with the bond valence method using the program VALENCE (Brown, 1996).

The indium ions are linked by the oxygen atoms of oxalate, giving rise to a three-dimensional interdependent porous framework (Fig. 3). The protonated ethylenediammonium and water molecules occupy the voids, interacting with oxalate anions through N—H···O and O—H···O hydrogen bonds. Without water molecules and cations, the framework exhibits voids possessing approximate dimensions of 6.9×14.5 Å along the crystallographic *c* axis and an analysis of the void shows that *ca* 44% of the space is empty. Thus, the ethylenediammonium and water molecules assigned to these cavities act as not only charge-compensating cations but also organic templates. The bond distances and angles for the bridging bidentate oxalate groups are in good agreement with the mean values reported by Hann (1957) for oxalate compounds, *i.e.*, 1.24 and 1.52 Å, 118 and 123° for the C1—O1 and C1—C2 bond lengths and O1—C1—C2 and O1—C1—O5 angles, respectively.

### Experimental

A mixture of Ti(SO_4_)_2_ (0.2 g, 0.84 mmol), H_2_C_2_O_4_.2H_2_O (1.0 g, 7.93 mmol), InCl_3_.4H_2_O (2 ml, 0.5 mol/*L*) and H_2_N(CH_2_)_2_NH_2_ (0.2 ml, CR) in H_2_O (5.0 ml) was sealed in a 20 ml stainless-steal reactor with Teflon liner and heated at 393 K for 2 days under autogenously pressure. Colorless crystals were isolated after the reaction solution was cooled gradually and washed with water.

### Refinement

H atoms bonded to C and N atoms were inlcuded in calcluated positions with C-H = 0.97 and N-H = 0.89Å and U_iso_(H) = 1.2U_eq_(C) or 1.5U_eq_(N). The H atoms bonded to O atoms were either included in calculated positions [O-H = 0.85Å] based on 'as found' locations or based on the most efficient H-bonding location and with U_iso_(H)= 1.0-1.2U_eq_(C).

### Crystal data

**Table:**

(C_2_H_10_N_2_)_0.5_[In(C_2_O_4_)_2_]·2H_2_O	*F*(000) = 2800
*M**_r_* = 357.95	*D*_x_ = 2.223 Mg m^−^^3^
Orthorhombic, *F**d**d*2	Mo *K*α radiation, λ = 0.71073 Å
Hall symbol: F 2 -2d	Cell parameters from 7228 reflections
*a* = 15.8498 (4) Å	θ = 2.6–27.9°
*b* = 31.1643 (8) Å	µ = 2.26 mm^−^^1^
*c* = 8.6618 (2) Å	*T* = 293 K
*V* = 4278.48 (18) Å^3^	Block, colourless
*Z* = 16	0.4 × 0.38 × 0.38 mm

### Data collection

**Table:**

Bruker SMART CCD diffractometer	1679 independent reflections
Radiation source: fine-focus sealed tube	1673 reflections with *I* > 2σ(*I*)
graphite	*R*_int_ = 0.025
φ and ω scans	θ_max_ = 25.0°, θ_min_ = 2.6°
Absorption correction: multi-scan (*SADABS*; Bruker, 2001)	*h* = −18→18
*T*_min_ = 0.426, *T*_max_ = 0.467	*k* = −36→36
7189 measured reflections	*l* = −10→10

### Refinement

**Table:**

Refinement on *F*^2^	Hydrogen site location: inferred from neighbouring sites
Least-squares matrix: full	H-atom parameters constrained
*R*[*F*^2^ > 2σ(*F*^2^)] = 0.018	*w* = 1/[σ^2^(*F*_o_^2^) + (0.0322*P*)^2^ + 7.4478*P*] where *P* = (*F*_o_^2^ + 2*F*_c_^2^)/3
*wR*(*F*^2^) = 0.046	(Δ/σ)_max_ = 0.001
*S* = 1.06	Δρ_max_ = 0.45 e Å^−^^3^
1679 reflections	Δρ_min_ = −0.71 e Å^−^^3^
160 parameters	Extinction correction: *SHELXTL* (Sheldrick, 2008), Fc^*^=kFc[1+0.001xFc^2^λ^3^/sin(2θ)]^-1/4^
13 restraints	Extinction coefficient: 0.00087 (5)
Primary atom site location: structure-invariant direct methods	Absolute structure: Flack (1983), 668 Friedel pairs
Secondary atom site location: difference Fourier map	Flack parameter: 0.00 (3)

### Special details

**Table:**

Geometry. All e.s.d.'s (except the e.s.d. in the dihedral angle between two l.s. planes) are estimated using the full covariance matrix. The cell e.s.d.'s are taken into account individually in the estimation of e.s.d.'s in distances, angles and torsion angles; correlations between e.s.d.'s in cell parameters are only used when they are defined by crystal symmetry. An approximate (isotropic) treatment of cell e.s.d.'s is used for estimating e.s.d.'s involving l.s. planes.
Refinement. Refinement of *F*^2^ against ALL reflections. The weighted *R*-factor *wR* and goodness of fit *S* are based on *F*^2^, conventional *R*-factors *R* are based on *F*, with *F* set to zero for negative *F*^2^. The threshold expression of *F*^2^ > σ(*F*^2^) is used only for calculating *R*-factors(gt) *etc*. and is not relevant to the choice of reflections for refinement. *R*-factors based on *F*^2^ are statistically about twice as large as those based on *F*, and *R*- factors based on ALL data will be even larger.

### Fractional atomic coordinates and isotropic or equivalent isotropic displacement parameters (Å^2^)

**Table:**

	*x*	*y*	*z*	*U*_iso_*/*U*_eq_	Occ. (<1)
In1	0.013694 (15)	0.314689 (7)	0.33266 (6)	0.01510 (11)	
O1	−0.06449 (17)	0.27864 (9)	0.1683 (4)	0.0277 (6)	
O2	0.10165 (19)	0.26861 (9)	0.1750 (4)	0.0290 (7)	
O3	0.04152 (19)	0.35822 (9)	0.1416 (3)	0.0241 (6)	
O4	0.14920 (17)	0.34173 (8)	0.3713 (3)	0.0220 (6)	
C1	−0.0334 (2)	0.25113 (11)	0.0817 (7)	0.0194 (7)	
C2	0.0620 (2)	0.24552 (11)	0.0823 (7)	0.0204 (7)	
C3	0.1128 (2)	0.37559 (10)	0.1357 (4)	0.0165 (7)	
C4	0.1740 (2)	0.36645 (11)	0.2696 (5)	0.0180 (8)	
O5	−0.07692 (18)	0.22769 (9)	−0.0061 (3)	0.0259 (7)	
O6	0.09025 (18)	0.21856 (9)	−0.0112 (4)	0.0302 (7)	
O7	0.13838 (16)	0.39927 (8)	0.0302 (3)	0.0223 (6)	
O8	0.24530 (18)	0.38498 (9)	0.2625 (4)	0.0231 (6)	
C5	0.2294 (3)	0.2715 (2)	−0.2277 (8)	0.0535 (16)	
H5C	0.1687	0.2676	−0.2275	0.064*	
H5A	0.2444	0.2864	−0.1332	0.064*	
N1	0.2527 (3)	0.29883 (17)	−0.3608 (6)	0.0502 (12)	
H1A	0.3035	0.2913	−0.3949	0.075*	
H1B	0.2534	0.3262	−0.3316	0.075*	
H1C	0.2150	0.2954	−0.4360	0.075*	
OW1	0.2500	0.2500	0.3570 (7)	0.0504 (16)	
HW1A	0.2938	0.2445	0.3040	0.050*	
OW2	0.0119 (6)	0.3117 (3)	−0.173 (3)	0.069 (2)	0.50
HW2B	0.0188	0.2848	−0.1847	0.083*	0.50
HW2A	0.0010	0.3173	−0.0793	0.083*	0.50
OW3	0.1418 (3)	0.36723 (19)	−0.2928 (7)	0.1000 (17)	
HW3B	0.1123	0.3788	−0.3632	0.120*	
HW3A	0.1542	0.3857	−0.2244	0.120*	

### Atomic displacement parameters (Å^2^)

**Table:**

	*U*^11^	*U*^22^	*U*^33^	*U*^12^	*U*^13^	*U*^23^
In1	0.01446 (16)	0.01545 (14)	0.01540 (15)	−0.00012 (8)	−0.00093 (14)	0.00069 (11)
O1	0.0185 (13)	0.0268 (13)	0.0378 (17)	−0.0013 (11)	0.0018 (14)	−0.0157 (13)
O2	0.0224 (15)	0.0289 (15)	0.0358 (17)	0.0008 (12)	−0.0078 (14)	−0.0104 (13)
O3	0.0187 (14)	0.0299 (14)	0.0237 (15)	−0.0058 (12)	−0.0031 (12)	0.0086 (11)
O4	0.0264 (15)	0.0220 (12)	0.0176 (15)	−0.0037 (10)	−0.0021 (11)	0.0065 (10)
C1	0.0209 (18)	0.0154 (15)	0.0218 (17)	−0.0017 (14)	0.001 (2)	0.0002 (16)
C2	0.0220 (19)	0.0194 (16)	0.0199 (17)	0.0002 (14)	0.000 (2)	−0.0018 (17)
C3	0.0177 (19)	0.0139 (15)	0.0179 (17)	0.0038 (13)	0.0005 (15)	0.0001 (14)
C4	0.019 (2)	0.0132 (15)	0.0214 (17)	0.0010 (14)	−0.0015 (16)	−0.0011 (14)
O5	0.0193 (15)	0.0251 (13)	0.0333 (17)	−0.0004 (11)	−0.0037 (13)	−0.0135 (13)
O6	0.0213 (15)	0.0324 (15)	0.0369 (19)	0.0024 (12)	0.0032 (14)	−0.0131 (14)
O7	0.0224 (13)	0.0229 (12)	0.0217 (14)	−0.0042 (10)	−0.0032 (11)	0.0066 (10)
O8	0.0180 (13)	0.0273 (15)	0.0239 (15)	−0.0057 (11)	−0.0039 (13)	0.0084 (12)
C5	0.031 (3)	0.080 (4)	0.049 (4)	−0.020 (2)	0.006 (3)	−0.016 (3)
N1	0.028 (2)	0.074 (3)	0.049 (3)	−0.006 (2)	−0.011 (2)	−0.001 (2)
OW1	0.026 (2)	0.086 (4)	0.039 (4)	0.024 (2)	0.000	0.000
OW2	0.084 (4)	0.067 (4)	0.055 (4)	0.013 (4)	−0.002 (5)	−0.006 (4)
OW3	0.083 (3)	0.146 (4)	0.072 (3)	0.016 (3)	−0.030 (3)	−0.045 (3)

### Geometric parameters (Å, °)

**Table:**

In1—O5^i^	2.168 (3)	C4—O8	1.270 (5)
In1—O3	2.185 (3)	O5—In1^iii^	2.168 (3)
In1—O1	2.196 (3)	O6—In1^iii^	2.370 (3)
In1—O8^ii^	2.230 (3)	O7—In1^iv^	2.327 (3)
In1—O7^ii^	2.327 (3)	O8—In1^iv^	2.230 (3)
In1—O4	2.331 (3)	C5—N1	1.480 (8)
In1—O6^i^	2.370 (3)	C5—C5^v^	1.492 (12)
In1—O2	2.423 (3)	C5—H5C	0.9700
O1—C1	1.242 (5)	C5—H5A	0.9700
O2—C2	1.248 (6)	N1—H1A	0.8900
O3—C3	1.253 (5)	N1—H1B	0.8900
O4—C4	1.235 (5)	N1—H1C	0.8900
C1—O5	1.260 (6)	OW1—HW1A	0.8500
C1—C2	1.522 (6)	OW2—HW2B	0.8502
C2—O6	1.250 (6)	OW2—HW2A	0.8500
C3—O7	1.243 (4)	OW3—HW3B	0.8498
C3—C4	1.539 (5)	OW3—HW3A	0.8500
			
O5^i^—In1—O3	140.63 (11)	C4—O4—In1	114.7 (2)
O5^i^—In1—O1	111.52 (10)	O1—C1—O5	123.2 (4)
O3—In1—O1	86.60 (12)	O1—C1—C2	118.2 (4)
O5^i^—In1—O8^ii^	92.22 (11)	O5—C1—C2	118.7 (4)
O3—In1—O8^ii^	96.82 (11)	O2—C2—O6	128.6 (4)
O1—In1—O8^ii^	137.63 (10)	O2—C2—C1	115.8 (4)
O5^i^—In1—O7^ii^	144.54 (11)	O6—C2—C1	115.5 (4)
O3—In1—O7^ii^	74.02 (11)	O7—C3—O3	125.5 (4)
O1—In1—O7^ii^	68.82 (10)	O7—C3—C4	117.2 (3)
O8^ii^—In1—O7^ii^	71.65 (10)	O3—C3—C4	117.3 (3)
O5^i^—In1—O4	72.66 (9)	O4—C4—O8	127.0 (4)
O3—In1—O4	72.43 (9)	O4—C4—C3	116.8 (3)
O1—In1—O4	142.91 (10)	O8—C4—C3	116.1 (3)
O8^ii^—In1—O4	76.46 (10)	C1—O5—In1^iii^	119.2 (3)
O7^ii^—In1—O4	129.79 (9)	C2—O6—In1^iii^	114.5 (3)
O5^i^—In1—O6^i^	71.78 (10)	C3—O7—In1^iv^	115.0 (2)
O3—In1—O6^i^	147.56 (11)	C4—O8—In1^iv^	118.0 (3)
O1—In1—O6^i^	75.75 (11)	N1—C5—C5^v^	114.0 (4)
O8^ii^—In1—O6^i^	79.52 (11)	N1—C5—H5C	108.7
O7^ii^—In1—O6^i^	74.28 (10)	C5^v^—C5—H5C	108.7
O4—In1—O6^i^	135.78 (10)	N1—C5—H5A	108.7
O5^i^—In1—O2	74.70 (10)	C5^v^—C5—H5A	108.7
O3—In1—O2	79.93 (11)	H5C—C5—H5A	107.6
O1—In1—O2	69.90 (11)	C5—N1—H1A	109.5
O8^ii^—In1—O2	152.36 (10)	C5—N1—H1B	109.5
O7^ii^—In1—O2	131.86 (10)	H1A—N1—H1B	109.5
O4—In1—O2	76.41 (9)	C5—N1—H1C	109.5
O6^i^—In1—O2	117.54 (10)	H1A—N1—H1C	109.5
C1—O1—In1	121.4 (3)	H1B—N1—H1C	109.5
C2—O2—In1	114.5 (3)	HW2B—OW2—HW2A	109.8
C3—O3—In1	118.7 (2)	HW3B—OW3—HW3A	109.8

Symmetry codes: (i) −*x*, −*y*+1/2, *z*+1/2; (ii) *x*−1/4, −*y*+3/4, *z*+1/4; (iii) −*x*, −*y*+1/2, *z*−1/2; (iv) *x*+1/4, −*y*+3/4, *z*−1/4; (v) −*x*+1/2, −*y*+1/2, *z*.

### Hydrogen-bond geometry (Å, °)

**Table:**

*D*—H···*A*	*D*—H	H···*A*	*D*···*A*	*D*—H···*A*
N1—H1C···OW1^vi^	0.89	2.35	2.880 (8)	118
N1—H1B···O7^iv^	0.89	2.47	2.956 (5)	115
N1—H1B···OW3	0.89	2.21	2.825 (8)	126
N1—H1C···O4^vi^	0.89	2.44	3.140 (6)	136
N1—H1C···O5^iii^	0.89	2.38	3.166 (5)	147
OW1—HW1A···O2^v^	0.85	2.04	2.889 (5)	180
OW2—HW2B···O1^iii^	0.85	2.46	3.241 (15)	153
OW2—HW2A···O3	0.85	2.39	3.12 (3)	145
OW3—HW3B···O8^vii^	0.85	2.19	2.870 (6)	137
OW3—HW3A···O7	0.85	2.26	2.971 (7)	141
OW3—HW3A···O3^iv^	0.85	2.40	2.962 (6)	124

Symmetry codes: (vi) *x*, *y*, *z*−1; (iv) *x*+1/4, −*y*+3/4, *z*−1/4; (iii) −*x*, −*y*+1/2, *z*−1/2; (v) −*x*+1/2, −*y*+1/2, *z*; (vii) *x*−1/4, −*y*+3/4, *z*−3/4.
